# The Influenza Virus H5N1 Infection Can Induce ROS Production for Viral Replication and Host Cell Death in A549 Cells Modulated by Human Cu/Zn Superoxide Dismutase (SOD1) Overexpression

**DOI:** 10.3390/v8010013

**Published:** 2016-01-08

**Authors:** Xian Lin, Ruifang Wang, Wei Zou, Xin Sun, Xiaokun Liu, Lianzhong Zhao, Shengyu Wang, Meilin Jin

**Affiliations:** 1 State Key Laboratory of Agricultural Microbiology, Huazhong Agricultural University, Wuhan 430070, China; yoya12@163.com (X.L.); wrf138493@126.com (R.W.); zouweixm@163.com (W.Z.); coolxin@foxmail.com (X.S.); a13886345180@sina.com (X.L.), zlzong12@163.com (L.Z.); sywang2012@126.com (S.W.); 2 Laboratory of Animal Virology, College of Veterinary Medicine, Huazhong Agricultural University, Wuhan 430070, China

**Keywords:** SOD1, H5N1, ROS, apoptosis, pro-inflamatory response, mitochondria

## Abstract

Highly pathogenic H5N1 infections are often accompanied by excessive pro-inflammatory response, high viral titer, and apoptosis; as such, the efficient control of these infections poses a great challenge. The pathogenesis of influenza virus infection is also related to oxidative stress. However, the role of endogenic genes with antioxidant effect in the control of influenza viruses, especially H5N1 viruses, should be further investigated. In this study, the H5N1 infection in lung epithelial cells decreased Cu/Zn superoxide dismutase (SOD1) expression at mRNA and protein levels. Forced SOD1 expression significantly inhibited the H5N1-induced increase in reactive oxygen species, decreased pro-inflammatory response, prevented p65 and p38 phosphorylation, and impeded viral ribonucleoprotein nuclear export and viral replication. The SOD1 overexpression also rescued H5N1-induced cellular apoptosis and alleviated H5N1-caused mitochondrial dysfunction. Therefore, this study described the role of SOD1 in the replication of H5N1 influenza virus and emphasized the relevance of this enzyme in the control of H5N1 replication in epithelial cells. Pharmacological modulation or targeting SOD1 may open a new way to fight H5N1 influenza virus.

## 1. Introduction

H5N1 avian influenza virus infections have spread worldwide and have caused remarkable economic and social impacts, which have raised serious worldwide concerns on a potential influenza pandemic. Infections with H5N1 viruses are associated with severe pneumonia, lymphopenia, high virus loads, and aggressively persistent trafficking of numerous inflammatory cells with hyper-induced cytokines and chemokines [1,2]. Viral infections, including Hepatitis (HCV), herpes simplex virus (HSV), respiratory syncytial virus (RSV), and influenza virus, occur simultaneously with the generation of excess reactive oxygen species (ROS) [3,4,5,6]; as a result, specific oxidant-sensitive pathways, such as mitogen-activated kinase p38, transcription factor nuclear factor-κB (NF-κB), and apoptosis, are activated [7,8,9,10,11]. H5N1-induced acute pulmonary damage can be attributed to excessive ROS production [12,13]. ROS possibly contributes to acute lung injury caused by severe influenza virus infection via the TLR4-TRIF-TRAF6 cascade by triggering oxidized phospholipid signaling [13].

ROS, including superoxide anion and its derivatives, are indiscriminately toxic to cells when these substances are produced excessively; ROS can also regulate pro-inflammatory responses. ROS is likely generated by inflammatory cells [14]. Lung epithelial cells are also a source of ROS because influenza virus can induce oxidative stress response in cultured airway epithelial cells [15,16]. Furthermore, numerous enzymes can generate superoxide [17], including those of the mitochondrial electron transport chain, nitric oxide synthase, cytochrome P_450_ reductases, and xanthine oxidase. However, for all of these systems, superoxide production occurs as a byproduct of another reaction or from a dysfunctional variant of the enzyme. NADPH oxidases are a family of multisubunit enzyme complexes that are unique in being the only enzymes indentified with the primary function of generating superoxide [18]. Currently, seven isoforms of NADPH oxidases (NOX) have been described in mammals [14]. Some of the isoforms have been reported to be related to influenza virus-induced lung inflammation and replication, such as NOX2 [19] and NOX4 [20]. ROS generation is generally a cascade of reactions that start with the production of superoxide, which then rapidly dismutates to hydrogen peroxide. The cascade of ROS generation also includes the reaction of superoxide with nitric oxide to form peroxynitrite, the peroxidase-catalyzed formation of hypochlorous acid from hydrogen peroxide, and the iron-catalyzed Fenton reaction leading to the generation of hydroxyl radicals [21]. ROS can avidly interact with a large number of molecules including other small inorganic molecules as well as proteins, lipids, carbohydrates, and nucleic acids. ROS may irreversibly destroy or alter the functions of the target molecule through such interaction. Thus, ROS have been increasingly considered as major contributors to damage biological organisms. However, various cellular enzymes, such as superoxide dismutases (SODs), catalase, and glutathione peroxidase, can participate in the defense against oxidative damage to maintain cellular redox homeostasis.

SODs are present in all cells in the body; these enzymes catalyze ROS detoxification and protect cells and tissues against oxidative stress. Extracellular superoxide dismutase (EC SOD) overexpression can prevent influenza-induced lung injury in mice by attenuating oxidative stress [22]. Cu/Zn superoxide dismutase (SOD1) was reported to be implicated in antioxidation in HCV-expressing cells [23]. SOD1 overexpression can reduce neurotoxic inflammatory signaling in microglial cells by altering ROS production [24]. It was also showed that SOD1 overexpression can inhibit H1N1 influenza virus replication in human alveolar epithelial cell line A549 [25]. Nevertheless, as H5N1 influenza viruses are more virulent agents that can lead to excessive cytokine secretion in infected mice and humans, it is of great interest to determine whether SOD1 overexpression can confer protection from H5N1 influenza viruses. This study demonstrates that H5N1 influenza virus infection can enhance ROS production to a greater extent than H1N1 influenza virus and that the former can induce intensive oxidative stress in A549 cells. Moreover, H5N1 infection increases the mRNA level of NADPH oxidases but reduces the mRNA and protein levels of SOD1. Forced SOD1 expression significantly decreases H5N1-induced ROS production and viral titer. This negative regulation of viral replication can be attributed to apoptosis inhibition, pro-inflammatory response attenuation, and protection against mitochondrial damage induced by H5N1 infection. Strategies targeting SOD1 may be an efficient way to control H5N1 infection.

## 2. Materials and Methods

### 2.1. Chemicals and Reagents

Dimethyl sulphoxide (DMSO) and diphenyleneiodonium (DPI) were obtained from Sigma–Aldrich (Saint Louis, MO, USA). Oxidant-sensitive dye 5-(and-6)-chloromethyl-2′,7′-dichlorodihydrofluorescein diacetate (CM-H2DCFDA) and MitoSox Red mitochondrial superoxide indicator purchased from Invitrogen (Carlsbad, CA, USA) were diluted in DMSO at 10 mM as stock. ATP-Lite Assay Kit was purchased from Vigorous Biotechnology (Beijing, China). ELISA Kit for IL-6 and TNF-α were purchased from Dakewe Biotech (Beijing, China).

### 2.2. Cells and Viruses

Human lung epithelial cell line A549 and Madin–Darby canine kidney (MDCK) cells were obtained from China Center for Type Culture Collection (Wuhan, China). Mouse primary lung epithelial cells were obtained from Rochen Biotech (Shanghai, China). A549 and primary mouse lung epithelial cells were kept in HAM’S/F-12 medium supplemented with 10% fetal bovine serum (FBS; HyClone, Logan, UT, USA). MDCK cells were grown in DMEM supplemented with 10% FBS. The cell cultures were incubated at 37 °C with 5% CO2. Influenza virus strains, including A/chicken/Hubei/327/2004 (H5N1) (DW) and A/PR/8/34 (H1N1) (PR8), were conserved by the State Key Laboratory of Agricultural Microbiology of China. These strains were propagated in nine-day-old specific pathogen-free embryonated eggs at 37 °C before use; the strains were then titrated by plaque assays in MDCK cells. For viral infections, DMEM or F-12 containing 1 μg/mL tolylsulfonyl phenylalanyl chloromethyl ketone (TPCK)-treated trypsin (Sigma-Aldrich, St. Louis, MO, USA) for H1N1 was used in MDCK or A549, respectively. For H5N1 virus infection, the medium was used without TPCK in trypsin. The experiments with H5N1 viruses were performed in a laboratory classified as Biosafety Level 3.

### 2.3. Antibodies

Rabbit polyclonal anti-IAV NP was kindly provided by Li Lin of the College of Fisheries, Huazhong Agricultural University, Wuhan, China. Mouse monoclonal anti-NP was conserved in our laboratory. Rabbit polyclonal Anti-SOD1, PCNA was purchased from ABclonal Biotechnology (Cambridge, MA, USA). Rabbit polyclonal anti-p38, p-p38, p65, p-p65, and cleaved caspase-3 were purchased from Cell Signaling (Beverly, MA, USA). Mouse monoclonal anti-GAPDH was purchased from California Bioscience (Coachella, CA, USA). Horseradish peroxidase (HRP)-conjugated anti-mouse and HRP-conjugated anti-rabbit secondary antibodies were purchased from SouthernBiotech (Birmingham, AL, USA). Alexa 488- or 594-conjugated goat anti-mouse or anti-rabbit secondary antibodies were obtained from Jackson ImmunoResearch (Newmarket, Suffolk, UK).

### 2.4. Plasmid Construction

Human SOD1 gene was obtained from cDNA of A549 cells through PCR. The fragment was then inserted into PCAGGS vector, which contains an HA tag in the N terminal. The promoter of the proximal region (−200 to +1) in SOD1 gene was cloned from genome of A549 cells, and inserted into PGL3-Basic vector (Promega, Fitchburg, WI, USA).

### 2.5. Biochemical Assays

The GSH/GSSH was evaluated from the total cell lysates of uninfected or infected cells at different post-infection time points by using GSH and GSSH assay kits (Beyotime, Shanghai, China) in accordance with the manufacturer’s instructions to determine the cellular antioxidant activity.

### 2.6. ROS Detection

The control cells and the infected A549 cells were washed twice with pre-warmed phosphate-buffered saline (PBS). Afterward, 5 μM CM-H2DCFA diluted in F-12 medium without FBS was added and incubated at 37 °C for 30 min. The cells were washed twice with PBS and were detached with 0.25% trypsin for FACS analysis by using the BD FACSCalibur system. For mitochondrial ROS detection, 5 μM MitoSox was used in the same way described above. At least 10,000 cells were analyzed, and the mean channel fluorescence intensity was calculated.

### 2.7. Apoptosis Detection

The control cells and the infected cells were washed with PBS, detached with 0.25% EDTA-free trypsin, and spun down. The cells were centrifuged at 1500 rpm for 5 min and were washed again with PBS. Cell apoptosis was then investigated by using an FITC-Annexin V apoptosis detection kit (Biolegand, San Diego, CA, USA) with the BD FACSCalibur system in accordance with the manufacturer’s instructions. At least 10,000 cells were analyzed. Caspase-3/7 activity was evaluated using a Caspase-Glo 3/7 assay kit (Promega) in accordance with standard instructions.

### 2.8. Real-Time Quantitative RT-PCR (qRT-PCR) Assay

The total RNA of differently treated cells was extracted using TRIzol (Invitrogen, Carlsbad, CA, USA) in accordance with standard instructions. Afterward, 1 μg of RNA was reversely transcribed. Gene expression was monitored through SYBR Green-based RT-PCR by using an ABI ViiA 7 PCR system (Apllied Biosystems, Foster City, CA, USA). Each gene expression was normalized to GAPDH. Significance was calculated using Student’s *t*-test, and *p* < 0.05 was considered statistically significant. The primers used in this study are listed in Table S1.

### 2.9. Western Blot

Differently treated cells were washed once with cold PBS and lysed in cell Tris lysis buffer (Cell Signaling) containing 1% EDTA-free protease inhibitor (Roche, Basel, Switzerland) in an ice bath for 30 min. The cells were then briefly sonicated and cleared through centrifugation at 12,000 rpm for 10 min at 4 °C. The supernatant was subsequently denatured by boiling for 5 min in SDS loading buffer. An equal amount of protein was subjected to SDS-PAGE and then transferred to nitrocellulose membranes (Whatman, Kent, UK). After the membranes were blocked in Tris-buffered Saline with Tween (TBST) containing 1% BSA, the membrane was incubated with various primary antibodies for 2 h at room temperature. The membrane was then washed thrice with TBST and reacted with the corresponding HRP-conjugated secondary antibodies for 1 h at room temperature. The membranes were washed again thrice; afterward, the blots were visualized using an Immobilon Western chemiluminescent HRP substrate kit (Thermo Fisher, Waltham, MA, USA) in an ECL detection system (Amersham Biosciences, Piscataway, NJ, USA).

### 2.10. Confocal Microscopy

A549 cells grown on coverslips were transfected with PCAGGS and PCAGGS-SOD1. After 24 h post-transfection, the cells were washed twice with F-12 medium without FBS. The cells were infected with five multiplicity of infection (MOI) of DW. After absorption was performed in an incubator with 5% CO_2_ for 1 h at 37 °C, the cells were washed twice. F-12-containing 0.5% FBS was added and incubated for 6 h. The cells were washed twice with PBS and fixed with 4% paraformaldehyde for 15 min. Afterward, the cells were permeabilized in 0.1% to 0.2% Triton X-100 for 10 min, and blocked with 1% BSA for 1 h at room temperature. The cells were incubated in PBS containing mouse anti-NP and rabbit anti-HA antibodies at 4 °C overnight. The cells were then incubated in PBS containing Alexa 488- and 594-conjugated goat anti-mouse or anti-rabbit secondary antibodies. After a final wash, the cells were stained with 4,6-diamidino-2-phenylindole (1 μg/mL in methanol) for 10 min. The fluorescence was visualized under Axiovert 200 confocal microscope (ZEISS, Oberkochen, Germany). Fifty cells expressing PCAGGS or PCAGGS-SOD1 were counted to determine the rate of NP nuclear export.

### 2.11. ATP Detection

SOD1- and control-expressed cells were infected with DW at 1 MOI or control to detect ATP. The cells were lysed at 24 hpi, and ATP concentrations were determined using an ATP-Lite assay kit (Vigorous Biotechnology, Beijing, China) in accordance with the manufacturer’s instructions. Results were presented as relative ATP concentration.

### 2.12. Determination of the Mitochondrial Membrane Potential (MMP)

The culture medium was changed to F-12 and was supplemented with 15 μM JC-10 (AAT Bioquest, Sunnyvale, CA, USA). After incubation was performed at 37 °C for 30 min, fluorescence was investigated using the BD FACSCalibur system.

### 2.13. Statistical Analysis

Data were expressed as mean ± SEM. Significance was determined by Student’s *t*-test, and *p* < 0.05 was considered statistically significant.

## 3. Results

### 3.1. H5N1 Infection Increased Cellular ROS Level in A549 Cells

**Figure 1:**
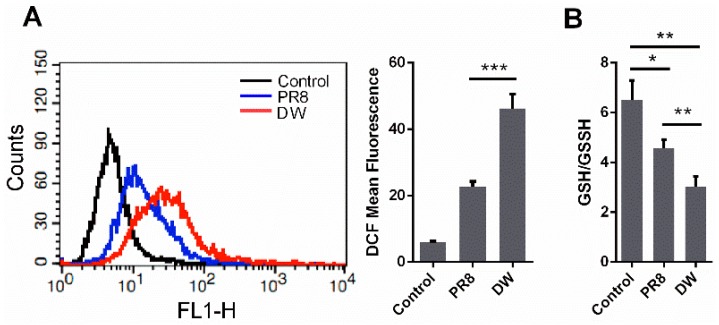
Highly pathogenic H5N1 influenza virus infection induced oxidative stress in A549 cells. (**A**) A549 cells were infected by H5N1 (DW), H1N1 (PR8), or control. 24 hpi, mean DCF fluorescence was determined via Flow cytometry as described in methods; (**B**) A549 cells were infected by DW, PR8, or control, 24 hpi, GSH/GSSH of whole cells was tested under manufacturer’s instruction. Data was mean ± SEM. Student’s *t*-test was used to analyze statistical significance. * *p* < 0.05, ** *p* < 0.01, *** *p* < 0.001.

A549 cells were infected with A/chicken/Hubei/327/2004 (H5N1) (DW) and PR8 at 1 MOI to determine the extent of intracellular ROS induced by influenza virus infection. Intracellular ROS levels were measured at 24 h post-infection (hpi). As shown in Figure 1A, both viruses could significantly increase intracellular ROS. However, DW induced more ROS than PR8. GSH/GSSH was evaluated to further investigate the intracellular redox state upon virus infection. The GSH/GSSG ratio is a key indicator of cellular antioxidant activities. In this study, the GSH/GSSG ratio in A549 cells remarkably decreased after H5N1 infection compared with that in the control cells (Figure 1B). By contrast, the reduction was not as drastic as that of H5N1, although H1N1 infection also diminished the GSH/GSSG ratio; the diminution was mainly due to the decrease of GSH, with no significant change in the level of GSSG. This finding was consistent with the reductions of intracellular ROS. Considering that the redox environment in A549 cells may be different from that in primary lung epithelial cells, we also investigated ROS production in mouse primary lung epithelial cells infected by H5N1 viruses (Figure S1). It showed that H5N1 viruses infection could also induce significant ROS production in the cells. The results suggested that H5N1 influenza viruses could induce intense ROS production, and could be stronger stimulating agents that boost intracellular ROS and induce oxidative stress than H1N1 viruses.

### 3.2. H5N1 Infection Modified the Expression of Oxidant and Antioxidant Enzymes

**Figure 2:**
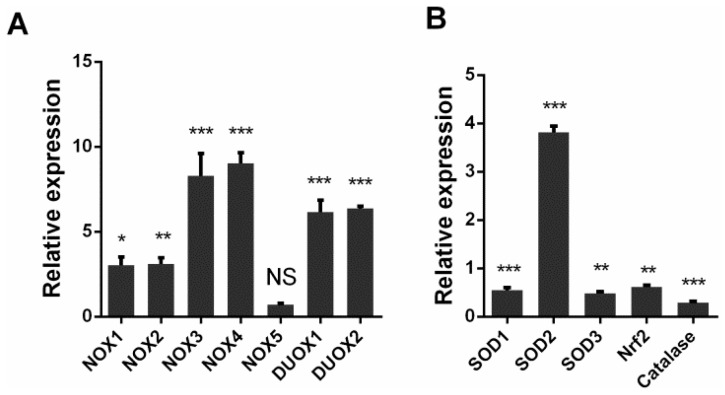
H5N1 infection promoted NADPH oxidases transcription, but downregulated key antioxidative genes transcription. A549 cells were infected by DW at 1 MOI, 24 hpi, total RNA was isolated for qRT-PCR. (**A**) NADPH oxidases transcription upon H5N1 infection. No-significant (NS) indicates *p* > 0.05; (**B**) some key antioxidative genes transcription upon H5N1 infection. GAPDH was used for normalization. Data was shown as relative expression to control infection and mean ± SEM of triplicate reactions. * *p* < 0.05, ** *p* < 0.01, *** *p* < 0.001.

To explore the mechanism responsible for the observed increase in intracellular ROS, we investigated the mRNA levels of NADPH oxidases and antioxidant enzymes through qRT-PCR. A549 cells were infected with DW at 1 MOI at 24 hpi, and total RNA was isolated for RT-PCR. The results showed that the mRNA of NADPH oxidases significantly increased in the H5N1-infected cells compared with that in the control cells, except NOX5 (Figure 2A). Several key antioxidant enzymes were also modulated by H5N1 infection. SOD1, SOD3, catalase, and Nrf2 were downregulated by viral infection. However, SOD2, which is mainly located in the mitochondria, significantly increased upon viral infection. The increase in NADPH oxidases combined with the depletion of SOD1, catalase, and Nrf2 may explain the accumulation of intracellular ROS in response to H5N1 infection.

### 3.3. SOD1 Disrupted H5N1 Replication in A549 Cells

**Figure 3:**
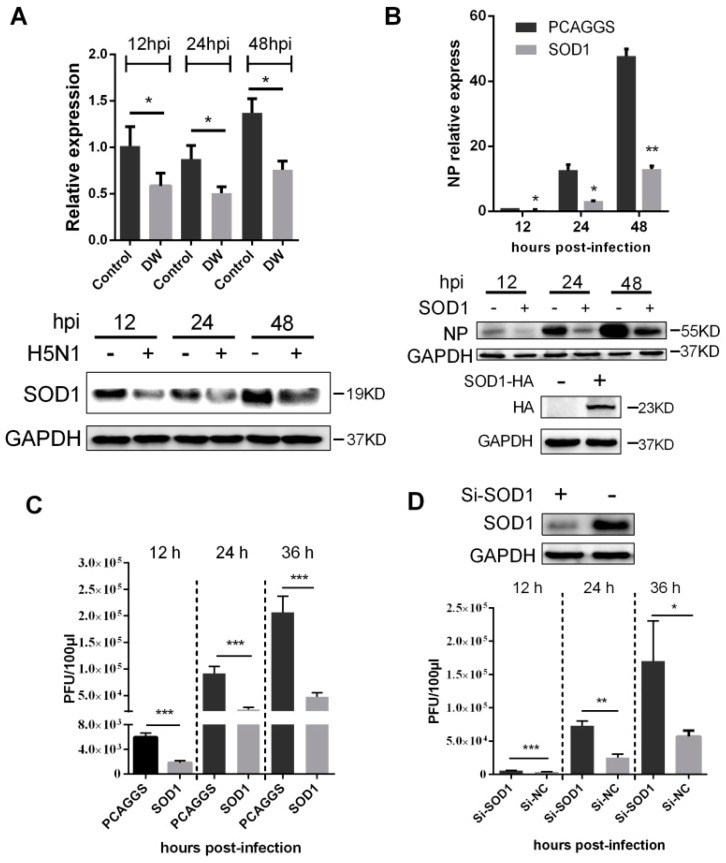
SOD1 overexpression inhibited H5N1 virus replication in A549 cells. (**A**) A549 cells were infected by DW or control at 1 MOI, and the mRNA of SOD1 and the protein were detected by RT-PCR (**up**) and western blot (**down**) at indicated time points; (**B**) SOD1 overexpressed A549 cells were infected by DW at 1 MOI, at different time post-infection, RNA and protein was prepared for NP detection by qRT-PCR (**up**) or western blot (**down**) respectively; (**C**) Cells were infected by DW at 2 MOI, at different time post-infecion, the supernatant was collected for virus titer test by plaque assay; (**D**) Si-RNA targeting human SOD1 was transfected in A549 cells, 36 h later, DW (2 MOI) was infected, at indicated time post-infection, the supernatant of NC or Si-RNA transfected cells upon virus challenge was collected for virus titer test by plaque assay at indicated time post-infection. NC: negative control of Si-SOD1 RNA. * *p* < 0.05, ** *p* < 0.01, *** *p* < 0.001.

Considering that SOD1 acts as a key scavenger to detoxify superoxide anion mainly found in the cytoplasm, we further investigated the changes in mRNA and proteins upon H5N1 challenge at different times post-infection. As shown in Figure 3A, the mRNA and the protein level of SOD1 significantly decreased as early as 12 hpi. The downregulation of SOD1 transcription may be due to the decreased promoter activity caused by virus infection (Figure S2). This observation prompted us to determine whether SOD1 depletion can contribute to H5N1 virus propagation. As shown in Figure 3B,C, mRNA, protein of viral nucleoprotein (NP), and released viruses titer significantly decreased in SOD1-overexpressed A549 cells compared with those of the control cells. mRNA of NP and released viruses were also remarkably inhibited in SOD1-overexpressed mouse primary lung epithelial cells (Figure S3). The virus replication significantly increased when the endogenous SOD1 was knocked down by siRNA transfection (Figure 3D). Overexpression or knock-down of SOD1 did not affect cellular viability (Figure S4). Furthermore, when A549 cells were pretreated with DPI, an inhibitor classically used to block ROS production, ROS level was decreased, and viral replication was significantly inhibited in a dose-dependent manner (Figure S5). These data together demonstrated that H5N1 virus infection could downregulate SOD1 expression, and SOD1 could be inversely related to H5N1 virus replication, which may be closely associated ROS production.

### 3.4. SOD1 Overexpression Reduced H5N1-Induced ROS Production and Attenuated Cytokine Response

**Figure 4:**
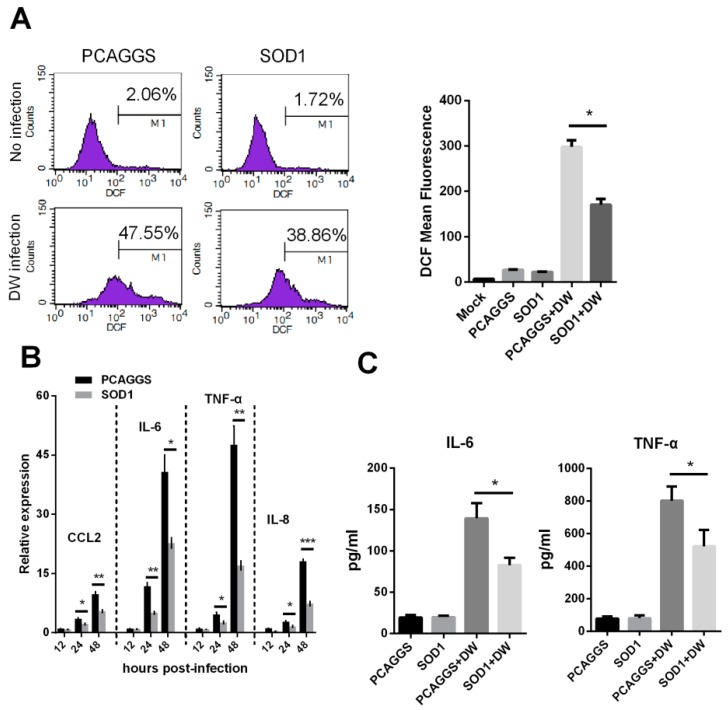
SOD1 overexpression attenuated intracellular ROS production and decreased pro-inflammatory response induced by H5N1 virus infection. (**A**) SOD1 or PCAGGS overexpressed A549 cells were infected by DW at 1 MOI, 24 hpi, cellular ROS level was investigated using H2-DCFDA via Flow cytometry; (**B**) Total RNA of SOD1 or PCAGGS overexpressed A549 cells infected by DW at indicated time post-infection was extracted for qRT-PCR analysis, and (**C**) IL-6 and IL-8 levels of supernatant were tested by ELISA. Data was mean ± SEM of triplicate reactions. * *p* < 0.05, ** *p* < 0.01, *** *p* < 0.001.

Considering that SOD1 acts as an important antioxidant enzyme, we investigated whether its overexpression can reduce H5N1-induced ROS production. Figure 4A and Figure S6 show that SOD1-overexpressed A549 cells and mouse primary lung epithelial cells produced significantly less ROS than the PCAGGS transfected cells. We then evaluated the effects of the SOD1 overexpression on pro-inflammatory response because H5N1 infection is often accompanied by the hyper-induction of cytokines. Figure 4B illustrates that the SOD1 overexpression significantly attenuated the transcription of several pro-inflammatory cytokines, including CCL2, IL-6, TNF-α, and IL-8. The protein level of IL-6 and TNF-α were also significantly decreased by SOD1 overexpression (Figure 4C).

### 3.5. SOD1 Overexpression Inhibited NF-κB and p38 Pathway and Disrupted the Nuclear Export of Viral NP

**Figure 5:**
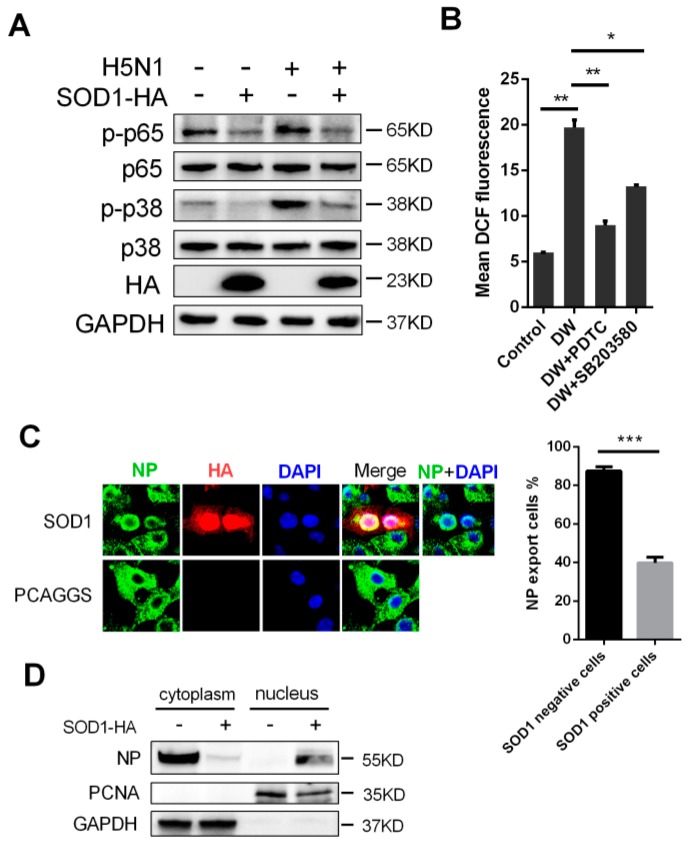
SOD1 overexpression inhibited p65 and p38 phosphorylation, and disrupted nuclear export of NP. (**A**) Control or DW-infected SOD1 overexpressed cells were lysed for indicated protein analysis using corresponding antibodies at 24 hpi; (**B**) Cells were pretreated with PDTC (NF-κB inhibitor) and SB203580 (p38 kinase inhibitor) with 100 and 5 μM respectively for 1 h followed by DW infection. At 24 hpi, DCF fluorescence was determined by Flow cytometry; (**C**) PCAGGS or SOD1 overexpressed A549 cells was infected by DW at 5 MOI, 6 hpi, cells were fixed, permeabilized, and reacted with corresponding primary and secondary antibodies. Fluorescence was viewed under a confocal microscope. NP export rate was calculated by counting at least 50 SOD1-positive or negative cells; (**D**) SOD1 or control transfected A549 cells were infected by DW at 5 MOI, 6hpi, protein of cytoplasm and nucleus were separated for indicated protein detection using corresponding antibodies. * *p* < 0.05, ** *p* < 0.01, *** *p* < 0.001.

NF-κB pathway is sensitive to oxidants and is implicated in influenza virus replication and pro-inflammatory response. In this study, A549 cells were infected with 1 MOI H5N1 to evaluate whether SOD1 can modulate the NF-κB pathway. The results showed that the SOD1 overexpression could significantly reduce the H5N1-induced phosphorylation of p65 (Figure 5A). The SOD1 overexpression also inhibited the p38 kinase pathway, which initiates the pro-inflammatory response to H5N1 infection, as the phosphorylation of p38 was significantly reduced by the SOD1 overexpression (Figure 5A). What was interesting was that inhibition of NF-κB and p38 kinase by the corresponding inhibitors significantly reduced the H5N1-induced intracellular ROS production (Figure 5B). These results indicated that SOD1 might negatively regulate H5N1-activated NF-κB and p38 pathways by eliminating ROS, which in turn impeded the activation of NF-κB and p38 pathways. Considering that the p38 kinase pathway is closely related to the nuclear export of virus ribonucleoprotein (RNP) complexes [26,27], we investigated the effect of the SOD1 overexpression on the retention of RNP complexes. A549 cells were overexpressed with SOD1 and infected with 5 MOI H5N1. The cells were analyzed in terms of RNP export at 6 hpi. The immunofluorescence staining results revealed that the nuclear export of the RNP complexes was inhibited in the SOD1-overexpressed cells (Figure 5C). Cytoplasm and nucleus separation assay also suggested its inhibition on NP export (Figure 5D).

### 3.6. SOD1 Overexpression Rescued H5N1-Induced Apoptosis

**Figure 6:**
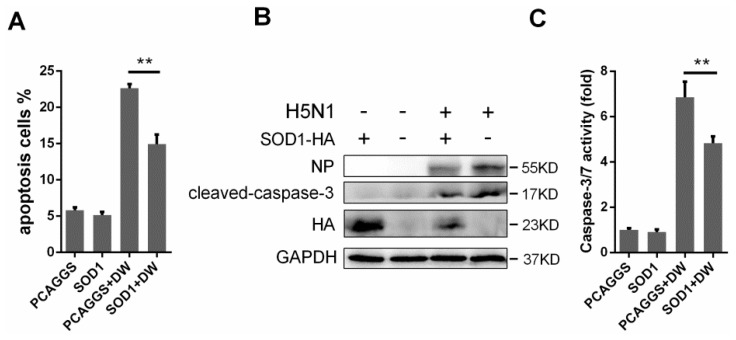
Forced SOD1 expression inhibited H5N1-induced cellular apoptosis. (**A**) A549 cells were infected by DW at 1 MOI, at 24 hpi, apoptosis was detected via Flow cytometry; (**B**) Western blots of cleaved-caspase-3 in SOD1 or control overexpressed A549 upon infection was detected; (**C**) caspase-3/7 activation was tested using Caspase-Glo 3/7 assay kit. Data was mean ± SEM. ** *p* < 0.01.

Oxidative stress is likely involved in virus-induced apoptosis [11,28]; as such, we investigated whether the ROS reduction by SOD1 overexpression can rescue H5N1-induced apoptosis. The control cells or the SOD1-overexpressed A549 cells were infected with DW at 1 MOI. The cells were collected, and the apoptosis rate was analyzed through flow cytometry. Figure 6A indicates that the SOD1 overexpression significantly reduced virus-induced apoptosis. Compared with that in the control cells, the SOD1 overexpression also decreased the cleavage of caspase-3 substrate (Figure 6B) and attenuated the activation of caspase-3/7 caused by H5N1 infection (Figure 6C). The apoptosis inhibition was also observed when the assay was performed in mouse primary lung epithelial cells (Figure S7). Additionally, the apoptosis was significantly decreased in DPI-treated cells (Figure S8). These results strongly suggested that the SOD1 overexpression can protect virus-infected A549 cells against apoptosis, and this may be related to alleviation of oxidative stress.

### 3.7. SOD1 Prevented Mitochondrial Dysfunction Caused by H5N1

**Figure 7:**
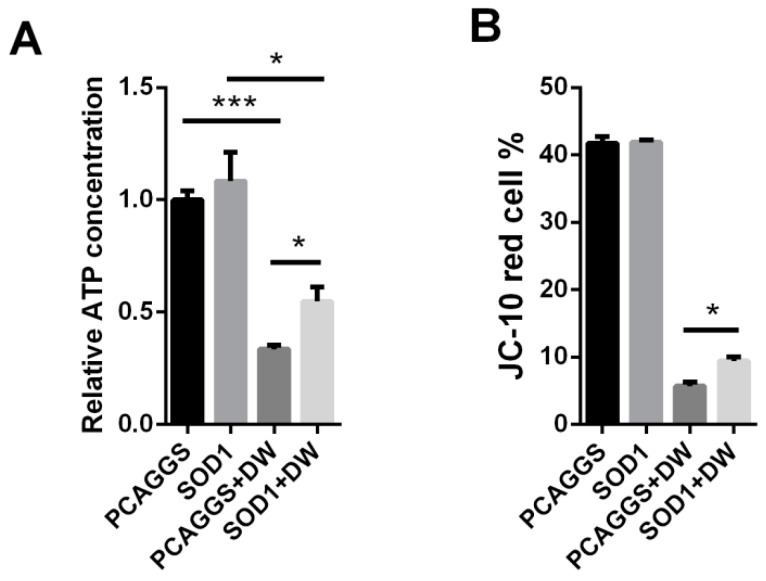

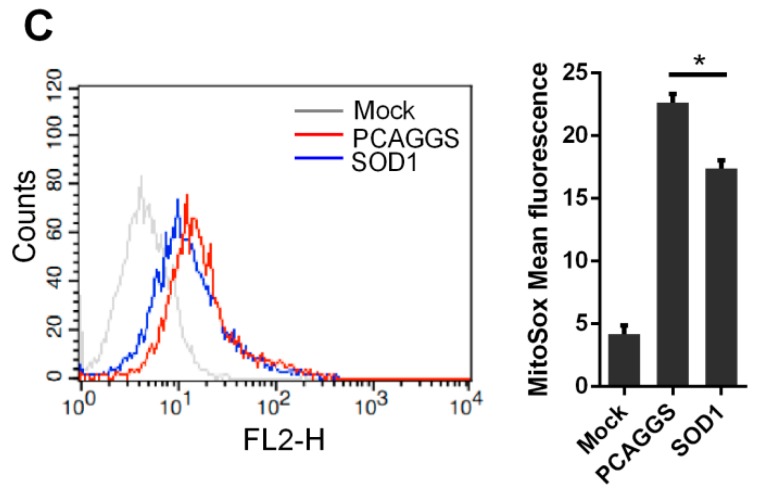
SOD1 overexpression prevented mitochondrial dysfunction caused by H5N1, SOD1, or PCAGGS transfected A549 cells were infected by 1 MOI DW, at 24 hpi, (**A**) cells were lysed for ATP detection; (**B**) MMP was investigated using 15 μM JC-10 by Flow cytometry; and (**C**) Mitochondrial ROS was tested using 5 μM MitoSox as described in methods. MitoSox mean fluorescence was calculated. * *p* < 0.05, *** *p* < 0.001.

Mitochondria play an important role in many cellular bioprocesses, such as ATP production, innate immune response, apoptosis control, and Ca^2+^ homeostasis regulation [29,30,31]. Mitochondria are also sources of ROS production and target of excessive ROS. In this regard, we evaluated the effects of the SOD1 overexpression on the mitochondrial function in H5N1-infected A549 cells. The H5N1 infection severely reduced the ATP production compared with the control infection in A549 cells (Figure 7A). The reduced level of ATP production in the virus-infected cells was remarkably lower than that in the control cells when SOD1 was overexpressed (Figure 7A). We also investigated whether the mitochondria membrane potential (MMP) is retained in SOD1-overexpressed A549 cells infected with H5N1 via JC-10 dye staining. Although the MMP in the SOD1-overexpressed cells was severely collapsed compared with that in the mock-infected cells, the extent of the reduction was inhibited compared with that of the control-transfected cells after H5N1 infection was induced (Figure 7B). Moreover, the SOD1 overexpression could attenuate the virus-induced mitochondrial ROS increase compared with that in the control cells, as determined by the mitochondrion-targeting superoxide indicator MitoSox (Figure 7C), which could weaken cellular oxidative stress and mitochondrial damage caused by mitochondrial ROS accumulation induced by H5N1 infection. These results suggested that SOD1 overexpression can prevent H5N1-induced mitochondrial dysfunction caused by H5N1 infection.

## 4. Discussion

Accumulating data demonstrate that viral infections can result in excess ROS production, including HCV, HSV, and RSV [3,4,5]. During influenza virus infection, ROS have often emerged due to disturbance of redox balance [32]. Studies from around the late 1980s suggest that ROS could promote lung injury and inflammation from influenza A virus infection [6,13]. Although organisms can be protected from severe influenza virus infection by targeting some major oxidases responsible for the ROS production via pharmacological strategies, such as the Nox2 oxidase inhibitor apocyanin [19], the effects of the modification of antioxidative enzymes on influenza virus replication remain largely unknown. Highly pathogenic H5N1 avian influenza virus infections are often accompanied with a high viral load and a hyper-induced pro-inflammatory response [1]. This behavior leads to increased mortality, which is observed to a greater extent in oxidative stress inducers than in other seasonal influenza viruses, such as H1N1. In the current study, H5N1 infection resulted in a significantly higher intracellular ROS than H1N1 infection in A549 cells, and remarkably decrease of GSH/GSSG compared with control. The decrease of GSH/GSSG, a key indicator of cellular antioxidant activities, was due to the significant reduction of GSH. GSH is found in almost every cell compartment, which acts as a redox buffer inside a cell [33]; GSH scavenges singlet oxygen and hydroxyl radicals, detoxifies hydrogen, and lipid peroxide and is a cofactor in several detoxifying enzymes [34]. Thus, GSH plays key role in protecting against oxidative stress. Previously, studies demonstrated that viruses infection can deplete host GSH and that the administration of exogenous GSH could inhibit viral replication [35,36,37]. Although the detailed mechanism of its antiviral activity needs to be further determined, it may be involved in inhibition of viral protein synthesis [38]. As to influenza virus, it is possible that higher levels of GSH could interfere with disulfide bond formation, and thus prevent the correct folding and mature of viral HA and consequently its transport and insertion into the cell membrane [39]. Besides, a recent study demonstrated that GSH-C4 strongly diminished influenza virus replication by detaining immature monomeric HA in the endoplasmic reticulum and reducing plasma-membrane targeting of mature glycoprotein, which is mediated by increased intracellular GSH levels [40]. It indicated that influenza virus infection could disrupt the balance of redox, and hijack the host antiviral strategy to benefit its replication, through modification of GSH. However, why influenza virus replication results in decrease of GSH remains unknown. It is possible that influenza virus infection could alter the rate of GSH biosynthesis or accelerate its efflux through unknown mechanism, which deserves further studies.

The relative balance of redox reactions is maintained in normal cells because of the well-developed antioxidant system, including enzymatic and non-enzymatic antioxidant systems, such as SODs and catalases. However, virus infection may modulate antioxidant systems and thus induce oxidative stress. For instance, respiratory syncytial virus can regulate antioxidant systems and stimulates oxidative stress [41]. In addition to the increase in NADPH oxidases caused by H5N1 infection in A549 cells (Figure 2A), the modulation of SOD1, SOD2, SOD3, Nrf2, and catalase was observed in A549 cells (Figure 2B). SOD1, SOD3, Nrf2, and catalase were downregulated by H5N1 infection; by contrast, SOD2 was upregulated by H5N1 infection. SOD1 is a detoxifying enzyme to convert superoxide radicals to molecular oxygen and hydrogen occurring in the cytoplasm; SOD3 is the dominant antioxidant enzyme found in a variety of extracellular compartments; Nrf2-antioxidant response element signaling pathway is a major mechanism in the cellular defense against oxidative stress [42]. The attenuation of the antioxidant cellular defenses, combined with the activation of ROS sources, possibly disrupts the pro-oxidant-antioxidant balance in favor of the former. SOD2 is mainly located in the mitochondria where this enzyme plays an important antioxidative role to protect the mitochondria from oxidative damage. The increased SOD2 expression by H5N1 infection indicated that different antioxidant genes may be modulated through various mechanisms upon H5N1 infection. The increased SOD2 expression may also be implicated in mitochondrial function upon H5N1 infection; however, this assumption should be further investigated.

In this study, SOD1 mRNA and protein level were downregulated by H5N1 virus infection. The proximal region (−157 to −25) of SOD1 gene contains many essential *cis*-acting elements for expression of SOD1. We found that H5N1 infection could decrease SOD1 promoter activity. This is in consistent with a previous study defining SP1 could be influenced by virus infection [25], which is a principal factor for SOD1 transcription [43,44]. However, we do not exclude the decreased protein of SOD1 is involved in post-translational levels, such as proteolysis by proteasome, which needs to be further determined. SOD1 can detoxify superoxide radical primary in the cytoplasm. Interestingly, it was reported to be implicated in antioxidant and antiviral activity in HCV-expressing cells [23], and reducing neurotoxic inflammatory signaling in microglial cells [24]. Nevertheless, the antiviral and antioxidative activities of SOD1 as a major antioxidant enzyme in H5N1-infected A549 cells remain unknown. We demonstrated in this study that the forced SOD1 overexpression could significantly disrupt H5N1 virus replication in A549 cells (Figure 3). This effect seems closely related to antioxidant activity because of the observed ROS reduction and decreased pro-inflammatory response in SOD1-overexpressed A549 cells stimulated by H5N1 compared with that in the control cells (Figure 4). ROS can activate oxidant-sensitive pathways, such as p38 and NF-κB, which are two important pathways that involve influenza virus replication and pro-inflammatory response. Although relatively active oxidative systems and attenuated antioxidative systems contribute to excessive ROS production to induce oxidative stress, some signaling pathways related to viral proliferation or cellular bioprocesses may also be potential promoters of oxidative stress. We can suppose that the attenuation of intracellular ROS by SOD1 could disrupt these pathways; in turn, the disrupted pathways can further decrease ROS production. NF-κB and p38 pathways are involved in influenza A virus pathogenesis. Thus, we evaluated the effects of SOD1 overexpression on the activation of these pathways upon H5N1 infection in A549 cells. Our results indicated that the SOD1 overexpression could weaken the H5N1-induced activation of both pathways. The treatment with specific inhibitors of NF-κB and p38 also significantly attenuated H5N1-induced ROS production in A549 cells. SOD1 may inhibit the H5N1 infection-activated NF-κB and p38 pathways in A549 cells via an unknown pathway, which is likely ROS elimination, to decrease virus-induced pro-inflammatory response; however, this finding should be further investigated. Influenza viruses pursue a nuclear replication strategy to export their genome to the cytoplasm in the form of a viral ribonucleoprotein (vRNP) complex from the nucleus. In particular, the p38 activation is necessary to initiate the nuclear export of viral vRNP [26,27], and this process is possibly under redox control [7]. We showed here that the accumulation of the vRNP was significantly inhibited in the SOD1-overexpressed cells compared with that in the control cells (Figure 5C). This finding may be closely related to antioxidant effects, which play a negative role in the p38 activation.

ROS is greatly associated with cellular apoptosis [11] and is likely responsible for the influenza virus-induced apoptosis through a caspase-dependent mechanism; ROS may also be involved in the NF-κB pathway [8,16]. Two major apoptosis signaling pathways involve upstream initiator caspases, such as caspase-8 and caspase-9, and downstream effector caspases, such as caspase-3 and caspase-7 [45,46]. Caspase-3 is an extensively investigated effector caspase and is considered as a major player in apoptosis regulation [47,48]. In our experiments, ectopic SOD1 overexpression could significantly decrease cellular apoptosis caused by H5N1 invasion. Moreover, caspase-3 cleavage and caspase-3/7 activation were downregulated by the SOD1 overexpression (Figure 6); this result suggested that SOD1 elicited an anti-apoptosis effect. In addition to apoptosis inhibition, the SOD1-induced inhibition of caspase-3 may be involved in the antiviral effect of SOD1 because caspase-3 is necessary to stimulate an efficient export of the influenza vRNP complex [49]. Interestingly, NF-κB pathway was likely inhibited by SOD1, as indicated by the detected p65 phosphorylation (Figure 5A). The SOD1 overexpression may exploit the NF-κB pathway to influence cellular outcomes upon influenza virus infection because the NF-κB pathway interference by N-acetyl-L-cysteine (NAC), a well-known antioxidant, can inhibit H5N1-induced apoptosis in A549 cells [50].

Mitochondria, which are an important ROS source in most mammalian cells, are a major component of apoptotic signaling pathways [31]. Cells undergo a decrease in the MMP caused by the opening of mitochondrial permeability transition (MPT), which is composed of voltage-dependent anion channel (VDAC) located in the outer mitochondrial membrane [51]. Proapoptotic proteins including Bak and Bax, can bind to VDAC and stimulate its opening and decrease of MMP [52]. Influenza virus could alter signal transduction pathway to decrease MMP. Previous study demonstrated that influenza virus PB1-F2 can bind to ANT3 and VDAC to open MPT [53]. The opening of MPT by PB1-F2 could further result in decrease of MMP. However, MPT opening can be also induced by generation of mitochondrial ROS, and another report showed the loss of MMP and generation of excess mitochondrial ROS were caused by activated caspases on mitochondrial electron transport complex I [54]. The mitochondrial ROS production is quiet controversial. The possible mechanisms involves deregulating mitochondrial enzymes, which impair electron transport, acceleration of Ca^2+^ cycling [54,55], and even the indirect effect of ROS production in cytoplasm due to its wide and complex activities. It is speculated that influenza virus may increase mitochondrial ROS production via regulating Ca^2+^ cycling, since that intracellular Ca^2+^ content can be regulated by influenza virus infection [56]. In this study, the forced SOD1 expression alleviated the mitochondrial dysfunction caused by H5N1 infection. It is possible that SOD1 overexpression can affect the signal pathway related to MPT opening and mitochondrial ROS production, because of the observed decrease of caspase 3 activity and ROS production. It seems that a close connection exists between cytoplasm-derived ROS and mitochondrial function, which is greatly attractive and needed to be further investigated. 

In all, although some targets and pathways responsible for ROS production were demonstrated to be relevant to influenza virus replication and the pathogenesis, less study was performed to focus on cellular targets for antioxidant that possibly disrupt influenza virus replication. This study described that SOD1 could inhibit the highly pathogenic H5N1 influenza virus, and this inhibition could be attributed to the antioxidant role of SOD1, which may attenuate virus-induced apoptosis, pro-inflammatory response, and mitochondrial dysfunction. Therefore, H5N1 infection may be effectively controlled by implementing strategies that target SOD1.

## 5. Conclusions

We demonstrated that H5N1 infection can down-regulate SOD1 expression. Forced SOD1 overexpression can disrupt H5N1 replication in A549 cells. This behavior may be attributed to the antioxidant role of SOD1; as a result, virus-induced apoptosis and pro-inflammatory response can be attenuated and H5N1 infection-induced mitochondrial dysfunction can be rescued to a certain extent.

## Supplementary Files

Supplementary File 1

## References

[1] De Jong M.D., Simmons C.P., Thanh T.T., Hien V.M., Smith G.J., Chau T.N., Hoang D.M., Chau N.V., Khanh T.H., Dong V.C. (2006). Fatal outcome of human influenza A (H5N1) is associated with high viral load and hypercytokinemia. Nat. Med..

[2] Perrone L.A., Plowden J.K., Garcia-Sastre A., Katz J.M., Tumpey T.M. (2008). H5N1 and 1918 pandemic influenza virus infection results in early and excessive infiltration of macrophages and neutrophils in the lungs of mice. PLoS Pathog..

[3] De Mochel N.S., Seronello S., Wang S.H., Ito C., Zheng J.X., Liang T.J., Lambeth J.D., Choi J. (2010). Hepatocyte NAD(P)H oxidases as an endogenous source of reactive oxygen species during hepatitis C virus infection. Hepatology.

[4] Pal S., Polyak S.J., Bano N., Qiu W.C., Carithers R.L., Shuhart M., Gretch D.R., Das A. (2010). Hepatitis C virus induces oxidative stress, DNA damage and modulates the DNA repair enzyme NEIL1. J. Gastroenterol. Hepatol..

[5] Gonzalez-Dosal R., Horan K.A., Rahbek S.H., Ichijo H., Chen Z.J., Mieyal J.J., Hartmann R., Paludan S.R. (2011). HSV infection induces production of ROS, which potentiate signaling from pattern recognition receptors: Role for *S*-glutathionylation of TRAF3 and 6. PLoS Pathog..

[6] Akaike T., Noguchi Y., Ijiri S., Setoguchi K., Suga M., Zheng Y.M., Dietzschold B., Maeda H. (1996). Pathogenesis of influenza virus-induced pneumonia: Involvement of both nitric oxide and oxygen radicals. Proc. Natl. Acad. Sci. USA.

[7] Torres M., Forman H.J. (2003). Redox signaling and the MAP kinase pathways. BioFactors.

[8] Gloire G., Legrand-Poels S., Piette J. (2006). NF-κB activation by reactive oxygen species: Fifteen years later. Biochem. Pharmacol..

[9] Asehnoune K., Strassheim D., Mitra S., Kim J.Y., Abraham E. (2004). Involvement of reactive oxygen species in toll-like receptor 4-dependent activation of NF-κB. J. Immunol..

[10] Brydon E.W., Morris S.J., Sweet C. (2005). Role of apoptosis and cytokines in influenza virus morbidity. FEMS Microbiol. Rev..

[11] Finkel T., Holbrook N.J. (2000). Oxidants, oxidative stress and the biology of ageing. Nature.

[12] Oda T., Akaike T., Hamamoto T., Suzuki F., Hirano T., Maeda H. (1989). Oxygen radicals in influenza-induced pathogenesis and treatment with pyran polymer-conjugated SOD. Science.

[13] Imai Y., Kuba K., Neely G.G., Yaghubian-Malhami R., Perkmann T., van Loo G., Ermolaeva M., Veldhuizen R., Leung Y.H., Wang H. (2008). Identification of oxidative stress and Toll-Like receptor 4 signaling as a key pathway of acute lung injury. Cell.

[14] Bedard K., Krause K.H. (2007). The NOX family of ROS-generating nadph oxidases: Physiology and pathophysiology. Physiol. Rev..

[15] Jacoby D.B., Choi A.M. (1994). Influenza virus induces expression of antioxidant genes in human epithelial cells. Free Radic. Biol. Med..

[16] Knobil K., Choi A.M., Weigand G.W., Jacoby D.B. (1998). Role of oxidants in influenza virus-induced gene expression. Am. J. Physiol..

[17] Droge W. (2002). Free radicals in the physiological control of cell function. Physiol. Rev..

[18] Selemidis S., Sobey C.G., Wingler K., Schmidt H.H., Drummond G.R. (2008). NADPH oxidases in the vasculature: molecular features, roles in disease and pharmacological inhibition. Pharmacol. Ther..

[19] Vlahos R., Stambas J., Bozinovski S., Broughton B.R., Drummond G.R., Selemidis S. (2011). Inhibition of NOX 2 oxidase activity ameliorates influenza a virus-induced lung inflammation. PLoS Pathog..

[20] Amatore D., Sgarbanti R., Aquilano K., Baldelli S., Limongi D., Civitelli L., Nencioni L., Garaci E., Ciriolo M.R., Palamara A.T. (2015). Influenza virus replication in lung epithelial cells depends on redox-sensitive pathways activated by NOX4-derived ROS. Cell Microbial..

[21] Thannickal V.J., Fanburg B.L. (2000). Reactive oxygen species in cell signaling. Am. J. Physiol. Lung Cell Mol. Physiol..

[22] Suliman H.B., Ryan L.K., Bishop L., Folz R.J. (2001). Prevention of influenza-induced lung injury in mice overexpressing extracellular superoxide dismutase. Am. J. Physiol. Lung Cell Mol. Physiol..

[23] Rivas-Estilla A.M., Bryan-Marrugo O.L., Trujillo-Murillo K., Perez-Ibave D., Charles-Nino C., Pedroza-Roldan C., Rios-Ibarra C., Ramirez-Valles E., Ortiz-Lopez R., Islas-Carbajal M.C. (2012). Cu/Zn superoxide dismutase (SOD1) induction is implicated in the antioxidative and antiviral activity of acetylsalicylic acid in HCV-expressing cells. Am. J. Physiol. Gastrointest. Liver Physiol..

[24] Dimayuga F.O., Wang C., Clark J.M., Dimayuga E.R., Dimayuga V.M., Bruce-Keller A.J. (2007). SOD1 overexpression alters ROS production and reduces neurotoxic inflammatory signaling in microglial cells. J. Neuroimmunol..

[25] Pyo C.W., Shin N., Jung K.I., Choi J.H., Choi S.Y. (2014). Alteration of Copper-Zinc superoxide dismutase 1 expression by influenza A virus is correlated with virus replication. Biochem. Biophys. Res. Commun..

[26] Pleschka S., Wolff T., Ehrhardt C., Hobom G., Planz O., Rapp U.R., Ludwig S. (2001). Influenza virus propagation is impaired by inhibition of the RAF/MEK/ERK signalling cascade. Nat. Cell Biol..

[27] Nencioni L., De Chiara G., Sgarbanti R., Amatore D., Aquilano K., Marcocci M.E., Serafino A., Torcia M., Cozzolino F., Ciriolo M.R. (2009). Bcl-2 expression and P38MAPK activity in cells infected with influenza A virus: Impact on virally induced apoptosis and viral replication. J. Biol. Chem..

[28] Shin N., Pyo C.W., Jung K.I., Choi S.Y. (2015). Influenza a virus PB1-F2 is involved in regulation of cellular redox state in alveolar epithelial cells. Biochem. Biophys. Res. Commun..

[29] Cheng Z., Ristow M. (2013). Mitochondria and metabolic homeostasis. Antioxid. Redox Signal..

[30] Dada L.A., Sznajder J.I. (2011). Mitochondrial Ca^2+^ and ROS take center stage to orchestrate TNF-α-mediated inflammatory responses. J. Clin. Investig..

[31] Kim R., Emi M., Tanabe K. (2006). Role of mitochondria as the gardens of cell death. Cancer Chemother. Pharmacol..

[32] Peterhans E., Grob M., Burge T., Zanoni R. (1987). Virus-induced formation of reactive oxygen intermediates in phagocytic cells. Free Radic. Res. Commun..

[33] Masella R., di Benedetto R., Vari R., Filesi C., Giovannini C. (2005). Novel mechanisms of natural antioxidant compounds in biological systems: involvement of Glutathione and Glutathione-related enzymes. J. Nutr. Biochem..

[34] Reshi M.L., Su Y.C., Hong J.R. (2014). RNA Viruses: ROS-mediated cell death. Int. J. Cell Biol..

[35] Ciriolo M.R., Palamara A.T., Incerpi S., Lafavia E., Bue M.C., de Vito P., Garaci E., Rotilio G. (1997). Loss of GSH, oxidative stress, and decrease of intracellular pH as sequential steps in viral infection. J. Biol. Chem..

[36] Garaci E., Palamara A.T., di Francesco P., Favalli C., Ciriolo M.R., Rotilio G. (1992). Glutathione inhibits replication and expression of viral proteins in cultured cells infected with Sendai virus. Biochem. Biophys. Res. Commun..

[37] Nucci C., Palamara A.T., Ciriolo M.R., Nencioni L., Savini P., D’Agostini C., Rotilio G., Cerulli L., Garaci E. (2000). Imbalance in corneal redox state during Herpes Simplex Virus 1-induced keratitis in rabbits. Effectiveness of exogenous glutathione supply. Exp. Eye Res..

[38] Nencioni L., Iuvara A., Aquilano K., Ciriolo M.R., Cozzolino F., Rotilio G., Garaci E., Palamara A.T. (2003). Influenza A virus replication is dependent on an antioxidant pathway that involves GSH and Bcl-2. FASEB J..

[39] Braakman I., Helenius J., Helenius A. (1992). Role of ATP and disulphide bonds during protein folding in the endoplasmic reticulum. Nature.

[40] Sgarbanti R., Nencioni L., Amatore D., Coluccio P., Fraternale A., Sale P., Mammola C.L., Carpino G., Gaudio E., Magnani M. (2011). Redox regulation of the influenza hemagglutinin maturation process: A new cell-mediated strategy for anti-influenza therapy. Antioxid. Redox Signal..

[41] Hosakote Y.M., Liu T., Castro S.M., Garofalo R.P., Casola A. (2009). Respiratory syncytial virus induces oxidative stress by modulating antioxidant enzymes. Am. J. Respir. Cell Mol. Biol..

[42] Nguyen T., Nioi P., Pickett C.B. (2009). The Nrf2-antioxidant response element signaling pathway and its activation by oxidative stress. J. Biol. Chem..

[43] Minc E., de Coppet P., Masson P., Thiery L., Dutertre S., Amor-Gueret M., Jaulin C. (1999). The human Copper-Zinc superoxide dismutase gene (SOD1) proximal promoter is regulated by SP1, EGR-1, and WT1 via non-canonical binding sites. J. Biol. Chem..

[44] Afonso V., Santos G., Collin P., Khatib A.M., Mitrovic D.R., Lomri N., Leitman D.C., Lomri A. (2006). Tumor necrosis factor-α down-regulates human Cu/Zn superoxide dismutase 1 promoter via JNK/AP-1 signaling pathway. Free Radic. Biol. Med..

[45] Wurzer W.J., Ehrhardt C., Pleschka S., Berberich-Siebelt F., Wolff T., Walczak H., Planz O., Ludwig S. (2004). NF-κB-dependent induction of tumor necrosis factor-related apoptosis-inducing ligand (TRAIL) and FAS/FASl is crucial for efficient influenza virus propagation. J. Biol. Chem..

[46] Cohen G.M. (1997). Caspases: The executioners of apoptosis. Biochem. J..

[47] Sanghavi D.M., Thelen M., Thornberry N.A., Casciola-Rosen L., Rosen A. (1998). Caspase-mediated proteolysis during apoptosis: Insights from apoptotic neutrophils. FEBS Lett..

[48] Slee E.A., Adrain C., Martin S.J. (1999). Serial killers: Ordering caspase activation events in apoptosis. Cell Death Differ..

[49] Wurzer W.J., Planz O., Ehrhardt C., Giner M., Silberzahn T., Pleschka S., Ludwig S. (2003). Caspase 3 activation is essential for efficient influenza virus propagation. EMBO J..

[50] Geiler J., Michaelis M., Naczk P., Leutz A., Langer K., Doerr H.W., Cinatl J. (2010). *N*-acetyl-l-cysteine (NAC) inhibits virus replication and expression of pro-inflammatory molecules in A549 cells infected with highly pathogenic H5N1 influenza a virus. Biochem. Pharmacol..

[51] Zamzami N., Susin S.A., Marchetti P., Hirsch T., Gomez-Monterrey I., Castedo M., Kroemer G. (1996). Mitochondrial control of nuclear apoptosis. J. Exp. Med..

[52] Shimizu S., Narita M., Tsujimoto Y. (1999). Bcl-2 family proteins regulate the release of apoptogenic cytochrome C by the mitochondrial channel VDAC. Nature.

[53] Zamarin D., Garcia-Sastre A., Xiao X., Wang R., Palese P. (2005). Influenza virus PB1-F2 protein induces cell death through mitochondrial ANT3 and VDAC1. PLoS Pathog..

[54] Ricci J.E., Gottlieb R.A., Green D.R. (2003). Caspase-mediated loss of mitochondrial function and generation of reactive oxygen species during apoptosis. J. Cell Biol..

[55] Richter C., Schlegel J. (1993). Mitochondrial calcium release induced by prooxidants. Toxicol. Lett..

[56] Ueda M., Daidoji T., Du A., Yang C.S., Ibrahim M.S., Ikuta K., Nakaya T. (2010). Highly pathogenic H5N1 avian influenza virus induces extracellular Ca^2+^ influx, leading to apoptosis in avian cells. J. Virol..

