# Supplementary material for: The Influenza Virus H5N1 Infection Can Induce ROS Production for Viral Replication and Host Cell Death in A549 Cells Modulated by Human Cu/Zn Superoxide Dismutase (SOD1) Overexpression
Source: Viruses. 2016 Jan 8;8(1):13. doi: 10.3390/v8010013 (PMC4728573; doi:10.3390/v8010013)
Supplement: Supplementary File 1 [file viruses-08-00013-s001.pdf]

Supplementary Materials: The Influenza Virus H5N1 Infection Can Induce ROS Production for Viral Replication and Host Cell Death in A549 Cells that Modulated by Human Cu/Zn Superoxide Dismutase (SOD1) Overexpression

Xian Lin, Ruifang Wang, Wei Zou, Xin Sun, Xiaokun Liu, Lianzhong Zhao, Shengyu Wang and Meilin Jin

Table S1. Primers used in RT-PCR.

Genes	Forward sequences	Reverse sequences
NOX1	CGCCCTTTGCTTCTATCTTG	AATTCCTCCATCTCCTGTTCC
NOX2	AAGATAGCGGTTGATGGGC	TTGAGAATGGATGCGAAGG
NOX3	CTAAGGACAATAGCAGGCGTCA	GCCAGGCTGAGAAAGAAGACG
NOX4	TTTTCCCAGGTAGGGTTTGT	CACATGATGTCCTTTGGTCTCA
NOX5	CTGGTGCCTGGAATCTTGTTTT	CTTGGAGGGGAGGAGGTTGA
DUOX1	GACAAGGATGGCAATGGCTAC	ATGCGGAACATAAGGCGAGAC
DUOX2	TGCCATCACCATCATTGCTCT	GCTGCTTCCTTCTTCACGCT
Nrf2	TCCAGTCAGAAACCAGTGGAT	GAATGTCTGCGCCAAAAGCTG
SOD1	TGGATCTGCCAACTACTCCC	CGTAGCCGAAGAAACCTCAT
SOD2	TGGACAAACCTCAGCCCTAA	TTGAAACCAAGCCAACCC
SOD3	CGCCTTCTTCGCCCTGGAG	TCAGGTCCCCGAACTGGTGC
Catalase	CTGTTGAAGATGCGGCGAGAC	TCCTGTGGCAATGGCGTTA
NP	CAGCGTTCAGCCCACTTTCT	GGGTTCGTTGCCTTTTCGTC
IL-6	AGGAGACTTGCCTGGTGAAA	CAGGGGTGGTTATTGCATCT
IL-8	TTGGCAGCCTTCCTGATTTC	TCTTTAGCACTCCTTGGCAAAAC
TNF-α	ACGGCATGGATCTCAAAGACAACC	TGAGATAGCAAATCGGCTGACGGT
CCL2	GCAATCAATGCCCCAGTCA	GCCTCTGCACTGAGATCTTCCT
GAPDH	GCTAAGGCTGTGGGCAAGG	GGAGGAGTGGGTGTCGCTG

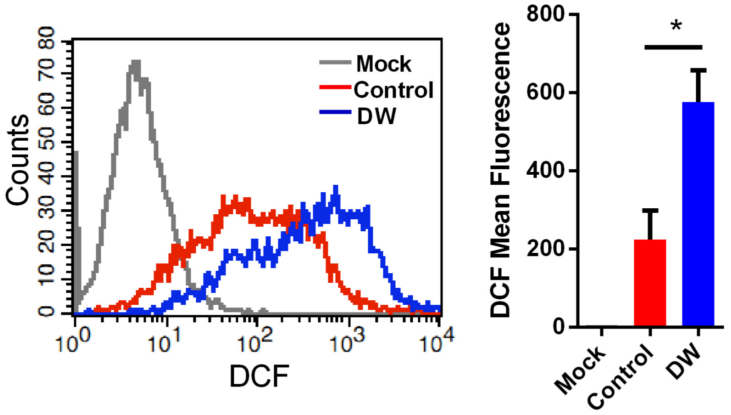

Figure S1. DW infection induced significant ROS production in mouse primary lung epithelial cells. Primary mouse lung epithelial cells were infected by DW at 1 MOI, 24 hpi, DCF mean fluorescence was detected by Flow cytometry. * $p < 0.05$.

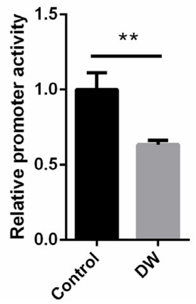

Figure S2. DW infection decreased SOD1 promoter activity. PGL3-basic vector containing proximal region (−200 to +1) of human SOD1 gene was transfected into A549 cells, 24 h after transfection, cells were infected by control or DW at 1 MOI. 12 hpi, fluorescence was detected using a dual-luciferase reporter assay system (Promega). ** $p < 0.01$.

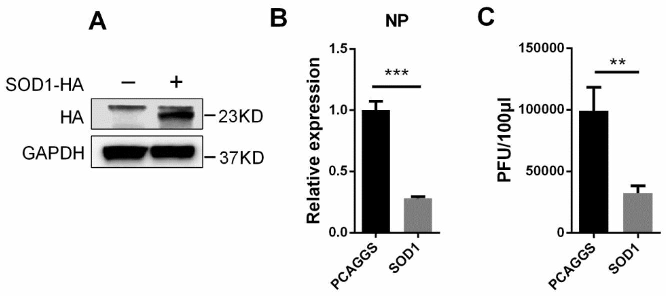

Figure S3. SOD1 overexpression inhibited viral replication in mouse primary lung epithelial cells. (**A**) SOD1 overexpression was verified 24 h after transfection; Primary mouse lung epithelial cells were infected by DW at 1 MOI, 24 hpi; (**B**) mRNA of viral NP was detected by qRT-PCR; and (**C**) viral titers in the supernatant were detected by plaque assay. ** $p < 0.01$, *** $p < 0.001$.

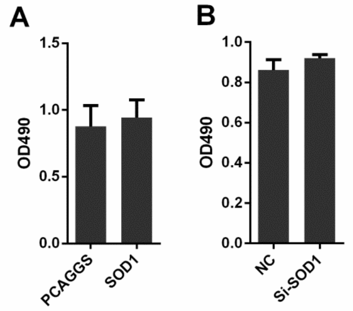

Figure S4. Cell viability test. A549 cells were transfected with PCAGGS or SOD1 (**A**) or siRNA (**B**), 36 h after transfection, cell viability was determined by MTT assay (Promega).

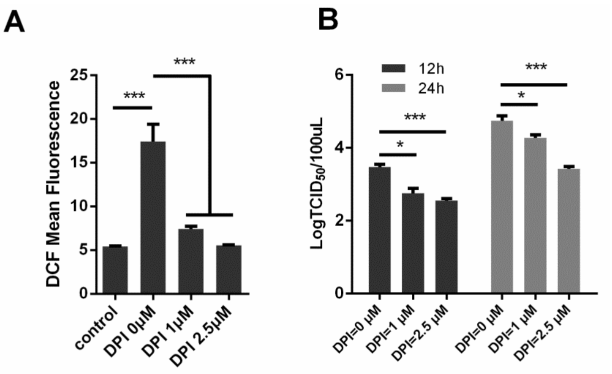

Figure S5. DPI inhibited virus induced ROS production and viral replication. A549 cells were pretreated with DPI, 24 hpi, (**A**) ROS production was detected by measuring DCF mean fluorescence via Flow cytometry; (**B**) viral titers were detected by $TCID_{50}$ assay. * $p < 0.05$, ** $p < 0.01$, *** $p < 0.001$.

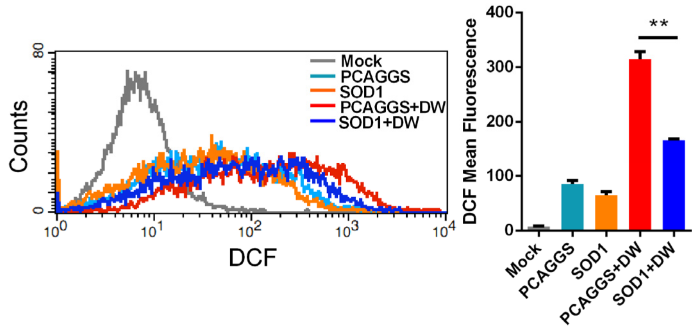

Figure S6. SOD1 overexpression inhibited ROS infection in mouse primary lung epithelial cells induced by DW infection at 1 MOI. ROS production was detected by measuring DCF mean fluorescence via Flow cytometry. ** $p < 0.01$.

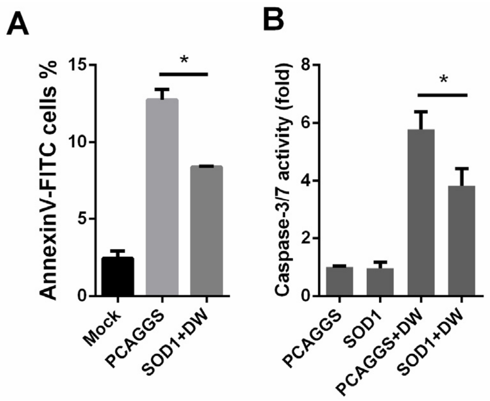

Figure S7. SOD1 overexpression inhibited virus induced apoptosis in mouse primary lung epithelial cells. Cells were infected by DW at 1 MOI, 24 hpi, (**A**) cells apoptosis was detected by Flow cytometry; (**B**) caspase-3/7 activity was tested using Caspase-Glo 3/7 assay kit. * $p < 0.05$.

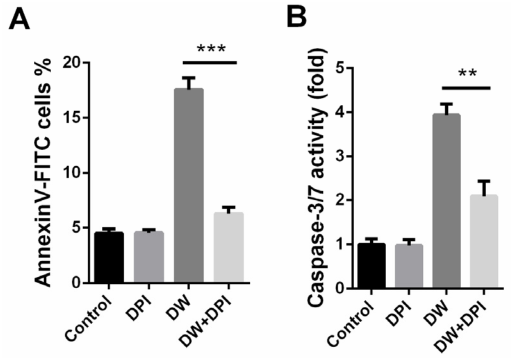

Figure S8. DPI inhibited virus induced apoptosis in A549 cells. A549 cells were pretreated with 2 μM DPI for 1 h, 24 hpi, (**A**) cells apoptosis was detected by Flow cytometry; (**B**) caspase 3/7 activity was tested using Caspase-Glo 3/7 assay kit. ** $p < 0.01$, *** $p < 0.001$.

© 2016 by the authors; licensee MDPI, Basel, Switzerland. This article is an open access article distributed under the terms and conditions of the Creative Commons by Attribution (CC-BY) license (http://creativecommons.org/licenses/by/4.0/).
